# High Adsorption of Benzoic Acid on Single Walled Carbon Nanotube Bundles

**DOI:** 10.1038/s41598-020-66871-4

**Published:** 2020-06-19

**Authors:** Shifan Li, Thushani De Silva, Iskinder Arsano, Dinuka Gallaba, Robinson Karunanithy, Milinda Wasala, Xianfeng Zhang, Poopalasingam Sivakumar, Aldo Migone, Mesfin Tsige, Xingmao Ma, Saikat Talapatra

**Affiliations:** 1 Zachry Department of Civil and Environmental Engineering, Texas A&M University, College Station, TX 77843 United States; 2 Department of Physics, Southern Illinois University Carbondale, IL 62901 Carbondale, United States; 3 Department of Polymer Science, University of Akron, Akron, Ohio 44325 United States

**Keywords:** Nanoscale materials, Theory and computation, Environmental impact, Surfaces, interfaces and thin films

## Abstract

Removal of harmful chemicals from water is paramount to environmental cleanliness and safety. As such, need for materials that will serve this purpose is in the forefront of environmental research that pertains to water purification. Here we show that bundles of single walled carbon nanotubes (SWNTs), synthesized by direct thermal decomposition of ferrocene (Fe(C_5_H_5_)_2_), can remove emerging contaminants like benzoic acid from water with high efficiencies. Experimental adsorption isotherm studies indicate that the sorption capacity of benzoic acid on these carbon nanotubes (CNTs) can be as high as 375 mg/g, which is significantly higher (in some cases an order of magnitude) than those reported previously for other adsorbents of benzoic acid such as activated carbon cloth, modified bentonite and commercially available graphitized multiwall carbon nanotubes (MWNTs). Our Molecular Dynamics (MD) simulation studies of experimental scenarios provided major insights related to this process of adsorption. The MD simulations indicate that, high binding energy sites present in SWNT bundles are majorly responsible for their enhanced adsorptive behavior compared to isolated MWNTs. These findings indicate that SWNT materials can be developed as scalable materials for efficient removal of environmental contaminants as well as for other sorption-based applications.

## Introduction

Carbon-based nanomaterials such as carbon nanotubes (CNTs) are actively explored by environmental scientists both as contaminants of emerging concern and as promising materials for a broad spectrum of nanotechnologies toward improved environmental outcomes^1–13^. The heightened interest in CNTs in environmental science derives primarily from their unique tubular structures, large specific surface area, tunable surface properties and strong adsorption capacity and affinity for a wide range of environmental chemicals. Such properties are often seen as very beneficial for a wide range of applications including the removal of environmental contaminants. Organic compounds represent a very large group of environmental contaminants and their molecular structures range from simple carbon-carbon aliphatics to multi-ringed aromatic structures with varying number of functional attachments. Many of these organic compounds are potent carcinogenic chemicals and are highly recalcitrant to biodegradation in the environment. Separation of these contaminants can be achieved through simple processes such as physical adsorption, which provides an effective means to control their bioavailability and alleviate their hazardous effects. In the past decade, a range of investigations on the adsorption behavior of CNT based-materials for a variety of chemical compounds/contaminants has been performed^12,13^.

In particular, the adsorption of emerging contaminants such as pharmaceuticals and personal care products have attracted significant attention recently^14,15^. Most of these contaminants contain aromatic rings and various functional groups. They are ionizable and therefore, often exist as a mixture of neutral and ionized compounds in the environment. The ionized compounds display unique adsorption mechanisms compared to their un-dissociated counterparts. Even though adsorption studies abound for both the neutral and ionized compounds, most studies follow a typical approach of calculating adsorption coefficients, and speculating mechanisms of adsorption through adsorption isotherm fitting. Detailed knowledge on the adsorption energy, the mechanisms of attachment, the adsorption kinetics and the role of water molecules at the molecular level is lacking. Only recently, molecular simulation is gradually being incorporated into adsorption studies which shed new light on the molecular level interactions at the liquid-solid interface^16,17^. Benzoic acid represents one of the model aromatic carboxylates used in a range of studies to investigate the adsorption of emerging contaminants on different environmental surfaces^18^.

Here we show that adsorption capacity of benzoic acid on single walled carbon nanotube (SWNT) bundles synthesized by direct thermal decomposition of ferrocene (Fe(C_5_H_5_)_2_)^19,20^ [and abbreviated as Fe-SWNT henceforth] is significantly higher than commercially available MWNTs. Results of our adsorption isotherm indicate that the sorption capacity of benzoic acid on these Fe-SWNT can be as high as 375 mg/g, which is about 5 times higher than those obtained for commercially available G-MWNT (~65 mg/g). Here we will like to note that, the specific surface area (SSA) of Fe-SWNT materials was ~2 times less than the reported SSA of the commercially available MWNT. Such enhanced adsorption of benzoic acid on SWNT materials could be due to their smaller tube diameters, as well as the availability of higher energy binding sites in the grooves of the bundles of Fe-SWNTs^21–24^. Groove sites are inherent to SWNT materials due to their tendency to form bundles during the synthesis process. In order to validate this reasoning, we performed detailed Molecular Dynamics simulation (MD) of the experimental scenarios. Simulated tube surfaces were found to selectively and effectively adsorb benzoic acid relative to the competing water. This was reflected in a several-fold difference in adsorption affinity between water and benzoic acid. A key insight from the MD simulations is that the grooves formed by SWNT bundles can offer high energy binding sites compared to isolated MWNTs and perhaps underlie the higher adsorption capacity of benzoic acids on SWNT as seen in our experiments. These findings indicate that SWNT materials can be developed for a variety of adsorption-based applications including the removal of environmental contaminants.

## Methods

### Sample synthesis

Typically the growth of carbon nanotubes using chemical vapor deposition is a combination of right thermodynamics along with the presence of metal catalyst and carbon source. In the present study, we have used ferrocene as both the catalyst (iron) as well as the carbon source for growth of the SWNTs. The basic growth mechanism in this particular case relies on the vaporization of the precursor to form the catalyst particle as well as the carbon vapors that react with the metal catalyst surfaces in order to form C-C bond resulting in the formation of the tubular structure of nanotubes. The Fe-SWNT were produced using direct thermal decomposition of ferrocene (Fe(C_5_H_5_)_2_) and detail description of the process is provided in our past publications^19,20^. This process is a single step production process to obtain networks of SWNT materials. The G-MWNT samples were obtained from U.S. Research Nanomaterials (Houston, TX). The G-MWNT synthesized by chemical vapor deposition (CVD) has a purity of > 95%, the inner and outer diameters of the G-MWNTs are 5–10 nm and 10–20 nm respectively. The length of the G-WMNT is 10–30 µm, with a specific surface area of ~200 m^2^/g according to the vendor^25^.

### Sample characterization

The as-produced materials were characterized using several experimental techniques. (a) Raman spectroscopy: The Raman spectra of the sample was acquired using iHR 550 (HORIBA Scientific). The excitation wavelength of the Raman spectrometer is 785 nm and it is equipped with an Olympus BX 41 microscope with 10×, 50×, and 100× magnification objectives. A 600 grooves/mm grating was utilized with laser power at 6 mW for the acquisition of data. Multiple Raman measurements were performed on several samples to ensure the reproducibility of the measurements. (b) X-Ray Photoelectron Spectroscopy (XPS): a Micron DAR 400 XPS was used to probe the surface chemical composition and bonding configuration of Fe-SWNTs, (c) Volumetric adsorption isotherm measurements: volumetric adsorption measurements for SSA determination was measured using nitrogen (N_2_) adsorption isotherm at liquid nitrogen temperatures (77 K). An automated adsorption setup (Micromeretics) was used for measuring the adsorption curves.

### Batch adsorption experiments

Benzoic acid was purchased from Fisher scientific (Houston, TX). Batch experiments were conducted for the adsorption of benzoic acid on Fe-SWNTs and G-MWNTs at pH 2.0 (at least two units below the pKa of benzoic acid to ensure they are not dissociated). To prepare benzoic acid solution, 1000 mg of benzoic acid solid was weighted and dissolved into 1000 mL deionized (DI) water. Solutions of benzoic acid at other concentrations (0.5–500 mg L^−1^) were obtained by serial dilution. The solution pH was then adjusted to around 2.0 with 0.1 N HCl. The precise value was measured with a pH meter and recorded. To maintain the distribution of benzoic acid between liquid and solid surface within a reasonable range (20–80%) so that the adsorbate would not disproportionally associate with only one phase, different solid liquid ratios were used for different initial concentrations of benzoic acid. Specifically, 5 mg of SWNTs or G-MWNTs were mixed with 10 mL solutions containing 0.5 and 5 mg L^−1^ benzoic acid in 20 mL brown vials; 10 mg CNTs were mixed with 10 mL of 50 and 200 mg L^−1^ of benzoic acid solution; and 15 mg CNTs were mixed with 10 mL of 500 mg L^−1^ benzoic acid in the same 20 mL brown vials. The mixtures were then sonicated for 8 hours in a bath sonicator (EMERSON, Houston, TX). Afterward, the mixtures were placed on a shaker table with 120 rpm for 7 days at ambient temperature (23 °C). The mixtures were then centrifuged at 3,000 rpm for 20 mins and the pH in the supernatant was measured again with a pH meter. The concentration of benzoic acid was analyzed with a Perkin Elmer UV-*vis*. spectrometer at the wavelength from 200 nm to 800 nm. The adsorbed benzoic acid was determined based on mass balance as reported in our earlier studies^12^.

### Molecular dynamics simulation

To achieve a computationally measurable adsorption, a 25% by mass benzoic acid (C_6_H_5_COOH) solution was equilibrated to dimensions of 16 nm by 16 nm by 8 nm at 298 K using the isothermal-isobaric (NPT) ensemble via the Nose-Hoover thermostat and barostat under periodic boundary conditions. A central cylindrical region was then removed from the system and in its place the desired carbon nanotube was inserted. Periodic boundary conditions were applied in all directions with an added vacuum buffer of 5 nm on the four lateral sides to approximate the adsorbed surface. The solution naturally curled around the carbon nanotube to minimize surface tension as the simulation progressed under constant volume and temperature (NVT) via the Nose-Hoover thermostat. Two kinds of nanotubes, a larger SWNT with chirality (40,40) and a smaller SWNT with chirality (10,10), were simulated for a total of 16 ns. In addition, tubes with chirality from (5,5) all the way up to (45,45), and graphene, were used for short tube/bundle-single molecule runs for a systematic assessment of curvature, bundle type, and groove size dependence of binding potential. The tubes were built using Visual Molecular Dynamics (VMD) software with Tachyon^26^. Visualization and image rendering were also performed using VMD. MD simulations were run using the Large-scale Atomic/Molecular Massively Parallel Simulator LAMMPS^27^ open-source software. Standard OPLS-AA force field^28^ parameters were combined with the Extended Single Point Charge (SPC/E) model^29^ for water for all bonded and non-bonded interactions. Solution preparation, and post analyses were completed using in-house scripts. Charge neutrality is maintained for all systems.

## Results and Discussion

**Figure 1:**
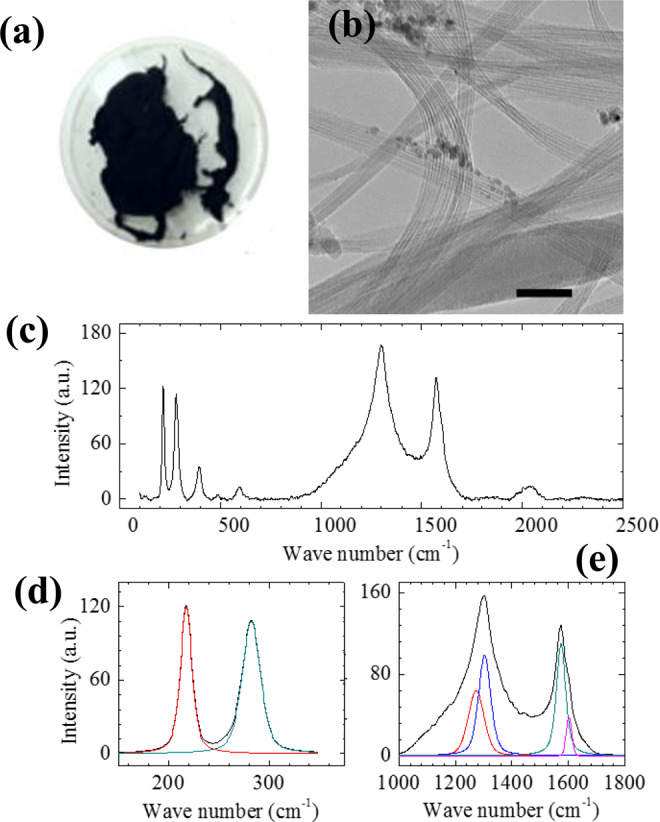
(**a**) Optical image of as produced Fe-SWNT materials in a 3 inch petri dish. (**b**) TEM image of bundles of Fe-SWNTs (scale bar = 50 nm). (**c**) Raman spectrum of the Fe-SWNT used in this study. (**d**) shows RBM data for Fe-SWNTs (details are in the text). (**e**) The D & G band region for the samples.

Raman spectroscopy is a powerful nondestructive material characterization technique, which is widely used for characterization of carbon-based materials in general and CNTs in particular. In Fig. 1 Raman spectroscopic measurements are presented. The most important Raman features related to Fe-SWNT are the radial breathing modes (RBM) and the D and G peaks (c to e in Fig. 1). While RBM can be utilized to calculate the nanotube diameters, the D and G peaks are related to the presence of disorder in graphene structure and the quality in nanotubes respectively. The diameter of the nanotubes corresponding to RBMs was calculated using the equation D = 248/ω where D is the diameter in nm and ω is the RBM frequency mode^30^. The Fe-SWNT sample shows higher intensities related to nanotubes with the diameters around 1.14 nm and 0.88 nm.

The intensity ratio between D (at 1303 cm^−1^) and G (at 1574 cm^−1^) bands (I_D_/I_G_) is found to be 1.26. This high D/G intensity ratio indicates the presence of disordered or defect/amorphous materials in our samples. We would like to note here that one of the key adsorption parameters that is affected by high D/G intensity ratio is the specific surface area available for adsorption. In other words, a carbon nanotube sample with high D/G ratio could have less specific surface area compared to purified carbon nanotube samples with low D/G ratio. However, influence of high D/G intensity ratio of binding energy of the adsorbents on a substrate (in our case carbon nanotubes) will have negligible effect. This is due to the fact that a clean ordered surface of carbon will offer different binding values to an adsorbate than its amorphous counterparts. Increasing the amount of ordered carbon base (low D/G ratio) will increase the availability of these higher binding sites (not their values) and hence an increase in specific surface area could be observed. In this particular study (see later sections for details) we conclude that the SWNTs are providing higher binding energy sites (due to bundling). These sites are available in the Fe-SWNT bundles irrespective of their D/G ratios.

The D band frequency (ω_D_) for SWNT bundles depend on the E_laser_ according to the equation ω_D_ = 1210 + 53 × E_laser_^30^. This equation implies that the D band peak is expected to appear at 1294 cm^−1^ for E_laser_ = 1.58 eV. As illustrated in Fig. 1(c) the actual D band was observed at 1303 cm^−1^, which is reasonably close to the calculated value. Since we are measuring the Raman spectroscopy for bundle SWNTs, the D band appears to have several peaks corresponding to different diameters of nanotubes. For isolated nanotubes, the D band frequencies should lie between 1286 to 1304 cm-1 for the laser with E_laser_ = 1.58 eV^30^. It appears that there exists another D band frequency at 1272 cm^−1^ (apart from the peak at 1303 cm^−1^) which is slightly off from the predicted range. The two peaks correspond to the D band that can appear from the semiconducting nanotubes present in our sample^30^. The peaks corresponding to G band lie between 1550–1605 cm^−1^^31^. The intermediate zone of the Raman spectra, which is from 500 cm^−1^ to 1000 cm^−1^ contains several peaks which correspond to overtones and combinations of lower frequency modes^32^.

**Figure 2:**
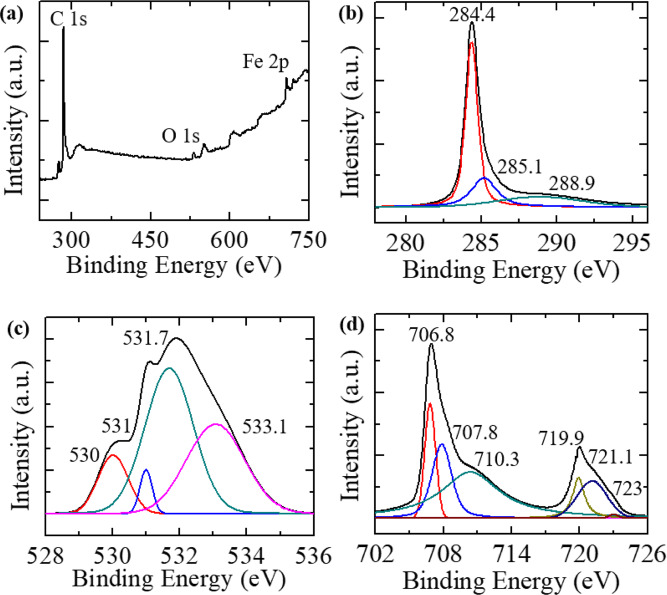
(**a**) XPS survey scan showing presence of C, O and Fe. (**b–d**) show decomposed peaks revealed through high resolution scans of the C1s, O1s and Fe2p, respectively.

We further performed XPS studies on Fe-SWNT samples in order to investigate their surface compositional characterization and bonding configuration on Fe-SWNT surface. The XPS scan results are presented in Fig. 2. In Fig. 2a, a typical survey scan is shown. This scan shows major peaks around 285 eV, 532 eV and around 707–720 eV. These peaks are generally associated with the carbon (C 1s), oxygen (O 1s) and iron (Fe 2p) respectively^33^. High resolution scans around these peaks to determine the nature of the bonding and surface chemical composition was performed. These scans for C, O and Fe are presented in Fig. 2b–d, respectively. The C1s spectrum can be decomposed into 3 peaks. The peak around 284.4 eV (which is slightly less than core carbon 1 s peak ~285 eV) can be assigned to C=C associated bonding type of highly oriented pyrolytic graphite (HOPG). This shows that the SWNT structures are well graphitized. Peaks around 285.1 eV can be due to the presence of C-OR type of functional groups. A weak broad peak ~288.9 eV typically signifies the presence of O-C=O (COOR type of functionalization) group. The surface O 1 s spectrum was decomposed into four peaks. The main O 1 s peak at ~531.7 eV is attributed to C-O bonds, and a broad shoulder ~533.1 eV could be assigned to O-C=O group. The presence of O-C=O group was also seen in C1s spectrum (as described above). The small peak at ~530.2 eV is characteristic of transition metal oxides and probably corresponds to a FeO, which might form during the CVD process and gets attached on the SWNT surface. Similarly, the small peak at ~531.1 eV can be assigned to Fe_2_O_3_ type of structure. The Fe spectrum was decomposed into six peaks. The peak around 706.8 eV is associated with Fe 2p3. The peak around 707.8 eV is perhaps due to the presence of some iron sulfide (FeS_2_) formed during the decomposition of ferrocene in the presence of sulfur. The peak at 710.3 eV is typically associated with γ-Fe_2_O_3_. Few peaks at higher values of binding energies were also observed. The peak around 719.9 eV is associated with Fe 2p1. The peak around 723 eV could be associated with FeO, but it could also come from Fe_3_O_4_, since, stoichiometric Fe_3_O_4_ can also be expressed as FeO.Fe_2_O_3_. From the above-mentioned analysis of the XPS data it was concluded that the Fe-SWNT samples have well graphitized carbon walls, containing oxides of iron.

### Specific surface area measurements

**Figure 3:**
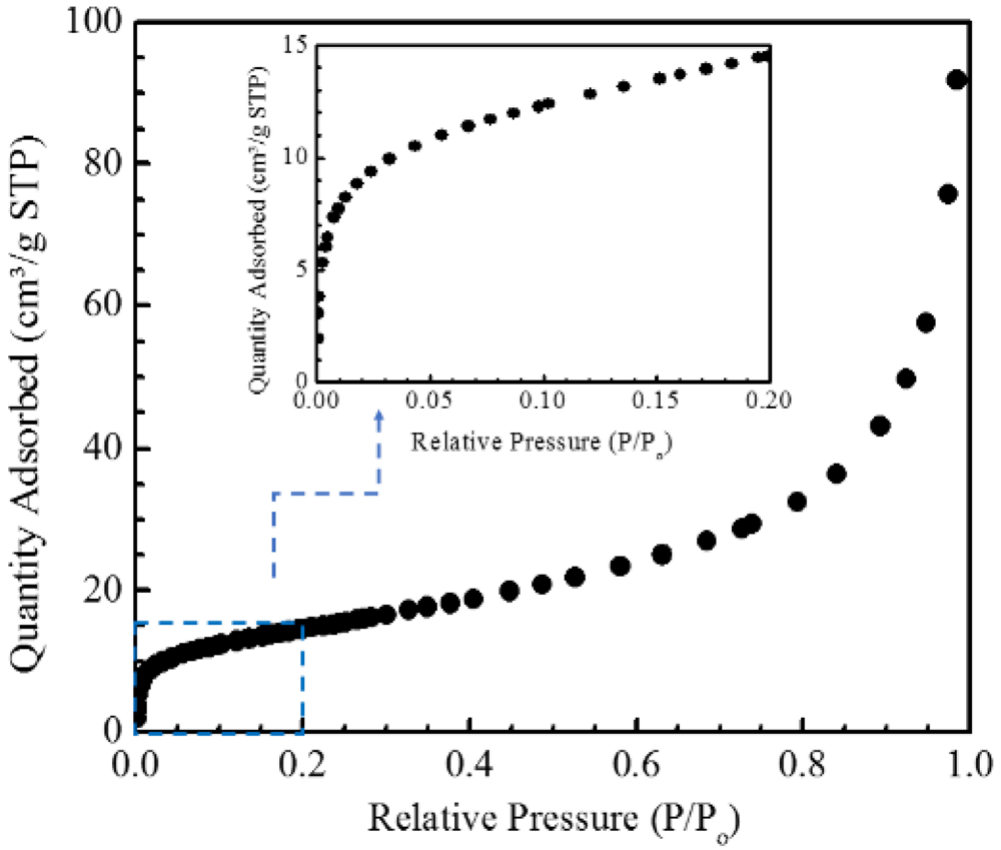
Volumetric adsorption isotherm data used for calculating the SSA. (Inset) The knee of the isotherm at lower coverages showing the completion of monolayer adsorption.

The SSA of the as produced Fe-SWNT was determined using Volumetric Adsorption Isotherm Measurement shown in Fig. 3. The adsorption isotherm data was used to determine the *Brunauer–Emmett–Teller* (*BET*) surface area of the samples^34^. A form of the equation is given as follows:

1 $$\frac{P/{P}_{o}}{n(1\,-\,P/{P}_{o})}=\frac{1}{C{n}_{m}}+\frac{(c\,-\,1)P/{P}_{o}}{C{n}_{m}}$$

where P is the pressure of the isotherm, P_o_ is the saturated vapor pressure of N_2_ at 77 K, n is the amount adsorbed at pressure P, n_m_ is the effective monolayer capacity, and C is a constant relating to the adsorbate/adsorbent strength of interaction. The SSA of the as produced Fe-SWNT was found to be ~52 m^2^/g. The samples also showed a broad pore size distribution with most of the pores in the meso (2–50 nm) to macro (>50 nm) pore range. Pore size distribution of typical Fe-SWNT samples are presented in the Supplementary Information (Fig. S1). This measured SSA is somewhat less than the reported values of SSA of SWNT’s produced by other techniques. One of the main reasons for such difference could be the fact that the direct thermal decomposition of metallocene (ferrocene in this case) will preserve some of the Fe in the form of oxides and/or sulphides either embedded in the CNT structures or on the surface, as evident from the XPS analysis. This causes the atomic weight percentage ratio of metal to carbon in such samples to be slightly higher.

### Adsorption of benzoic acid on SWNTs

The pKa of benzoic acid is 4.76, therefore, at the tested pH (pH = 2.0), benzoic acid is fully protonated. We found that benzoic acid generally displayed good adsorption capacity on the fully graphited G-MWNTs. This is expected due to the nanoscale dimension of the G-MWNTs as well as their reported BET specific surface area value of ~200 m^2^/g. It is also well known that hydrophobic effects, van der Waals forces and π-π interactions are important for the adsorption of aromatic compounds to CNTs. Addition of functional groups such as O-containing functional groups on CNT surface have complex and sometimes opposing effects on the adsorption of environmental pollutants, depending on the polarity of concerned chemicals. On one hand, they tend to decrease the hydrophobicity of the CNT surface and weaken π-π interactions, resulting in lower adsorption of hydrophobic compounds^35^. On the other hand, O-containing functional groups increase the hydrophilicity of CNTs, resulting in greater dispersion in solution and more available sites for adsorption. Since our MWNT’s were graphitized, impact of functionalization on the adsorption of benzoic acid can be ignored. We estimated the maximum adsorption capacity of benzoic acid on G-MWNT by fitting the adsorption data with Langmuir Isotherm equation given below^18^:

2 $${q}_{e}={q}_{max}\frac{b{C}_{e}}{1\,+\,b{C}_{e}}$$

where q_e_ is the adsorption capacity at equilibrium, q_max_ is maximum adsorption capacity, C_e_ is the equilibrium concentration of the adsorbate, and b is the Langmuir adsorption constant. We find that the q_max_ value for G-MWNT is ~65 mg/g and is similar to the adsorption capacity of other reported adsorbents such as modified bentonite and vermiculite^36^. The estimated b value was around 0.006 L/mg.

**Figure 4:**
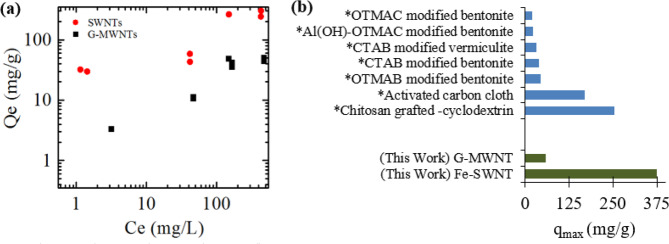
(**a**) Adsorption of benzoic acid on commercially available G-MWNT and Fe-SWNT. (**b**) Comparison of maximum adsorption of G-MWNT and Fe-SWNT with other reported adsorbents for benzoic acid. *indicates data obtained from ref. ^38^ and references therein.

Next, we examined the adsorption capacity of benzoic acid on Fe-SWNT and compared it with other adsorbents. These are presented in Fig. 4a,b respectively. From our XPS and volumetric adsorption measurements on Fe-SWNT it was clear that, although very low, there are some signs of surface functionalization on Fe-SWNT. SSA value ~52 m^2^/g (Note: this specific SSA is at least 2 times *less* than the G-MWNT) was registered. Given the low SSA and indication of functionalization, it can be generally hypothesized that the adsorption capacity of Fe-SWNT will be small, since both low SSA and functionalization should lead to less adsorption capacity. However, our results are starkly different from this general expectation. Our experimental results indicate that the adsorption of benzoic acid on Fe-SWNT was significantly higher than G-MWNTs. Data comparison of the adsorption of benzoic acid on G-MWNT and Fe-SWNT on log scale is shown in Fig. 4a (we have presented this data in linear scale along with the Langmuir fitting in Supplementary Information Fig. S2). We have also estimated the maximum adsorption capacity, q_max_, of benzoic acid on Fe-MWNT by fitting the data with Langmuir Isotherm (similar to the analysis followed while estimating the q_max_ of G-MWNT). We found that the q_max_ values are ~ 375 mg/g, which is significantly higher than that of G-MWNT as well as several other adsorbents that are investigated in the past^37,38^. The estimated b value from Langmuir fitting for Fe-SWNT was only 28.6% of the value estimated for G-MWNTs (0.021 L/mg). The lower b value for Fe-MWNTs suggests that Langmuir model, a surface adsorption-based model, did not fully capture the adsorption mechanism of benzoic acid on Fe-SWNTs, which as our simulation study demonstrated, involves adsorption to the high energy groove sites between nanotubes.

Although the availability of high SSA is considered one of the most dominant parameters governing the adsorption capacity of environmental pollutants on environmental surfaces, here we can see that this is not the case for Fe-SWNT. For example, the estimated q_max_ for Fe-SWNT is about 2.3 times higher than that on activated carbon cloth with a massive SSA of 2500 m^2^/g^33^. The q_max_ is almost a magnitude higher than a G-MWNTs with a SSA of 117 m^2^/g reported in the literature^17^. Several published reports affirm that the bundling of SWNTs can provide additional higher binding energy sites for adsorption in the grooves formed by two adjacent tubes in a bundle in comparison to isolated MWNT. The substantially higher q_max_ of benzoic acid on Fe-SWNTs with smaller SSA compared to other reported substrates suggests that high-energy binding sites that are present in Fe-SWNT bundle structures are perhaps accessible to benzoic acid and are energetically favorable for their adsorption. These observations are largely in accord with the mass density and adsorption potential parameters computed via MD simulations and discussed in the following section.

### Molecular Dynamics (MD) simulations

The effectiveness of CNTs for the targeted adsorption of benzoic acid as a model contaminant is investigated with MD simulation of CNTs of two different radii (chirality (10,10) with r = 6.77 ${\rm{\AA }}$, and chirality (40,40) with r = 27.08 ${\rm{\AA }}$, simulating a SWNT and a MWNT, respectively. The simulations are designed to approximate exterior adsorption on capped CNTs.

### Mass adsorption

**Figure 5:**
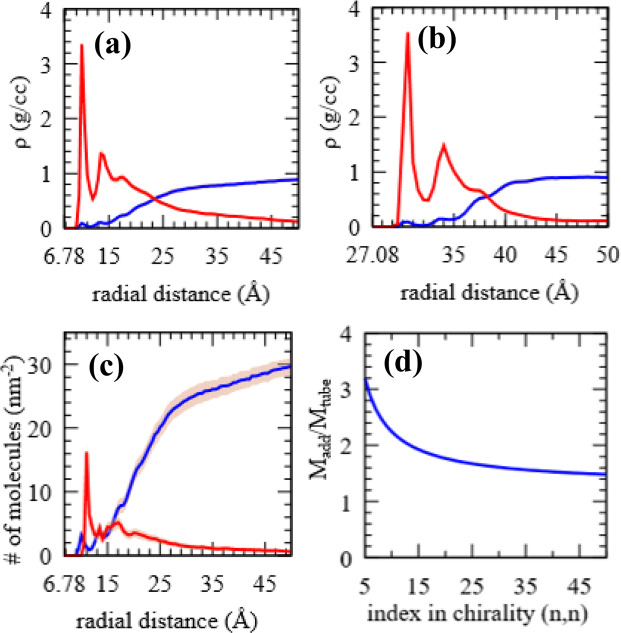
(**a**,**b**) Mass density curves surrounding CNT (10,10) and CNT (40,40), respectively. (**c**) Number density per 1 nm radial thickness and for every nm^2^ of CNT (10,10) surface area. Benzoic acid in red, and water in blue; error margins shown by the shaded regions; curves shown from tube surface. (**d**) Proportion of adsorbent mass to tube mass (M_ads_/M_tube_) for typical armchair nanotubes plotted with respect to increasing tube diameters represented by respective chrialties. Adsorption thickness and average density in adsorption region were maintained at $10\,{\rm{\AA }}$ and $1g/cc$, respectively, to establish a qualitative trend in (**d**).

In order to determine the mass adsorption profile, adsorption runs were performed for a total of 16 ns and with up to four replacement cycles. Each replacement cycle is initiated when the region surrounding an adsorption shell is fully depleted. Such a depletion results in lack of ready adsorbents that can bind to tube that is not fully saturated. The height of peaks expectedly decreases with radial distance. The emergence of a third adsorption peak signifies two fully saturated adsorption shells. $r=16\,{\rm{\AA }}$ in Fig. 5a, and $r=36\,{\rm{\AA }}\,{\rm{in}}$ Fig. 5b are taken as the respective adsorption region cutoff distances. The final mass and number density curves for the small tube are presented. Mass adsorbed per mass of the tube (M_ads_/M_tube_) was also estimated. We found that the total mass of benzoic acid adsorbed on the small tube is $1845.4\pm 14.7\,mg/g$ while that for the large tube is $1152.9\pm 6.5\,mg/g$. The adsorption regions for the two cases are comparable at close to $10\,{\rm{\AA }}$ in thickness. Average densities within the respective cylindrical shells fall at $0.86g/cc$ and $0.80\,g/cc,$ respectively for the large and small tubes. The region farther out from the tube is a solute-depleted region occupied by water that has nearly attained its bulk density of $1g/cc$. Number density per 1 nm radial thickness and for every nm^2^ of CNT (10,10) surface area is shown in Fig. 5c. Maintaining the adsorption thickness at $10{\rm{\AA }}$ and average shell density at $1g/cc$ a general qualitative trend can be established whereby smaller tubes have an advantage in retaining more adsorbent per unit mass of tube (Fig. 5d).

### Binding potential

Binding potential calculations were performed using multiple 1 ns simulations involving a single benzoic acid allowed to adsorb on differently sized multi-walled and aggregated tubes. The strength of adsorbent – tube interaction is characterized by a 12/6 Lennard-Jones potential between adsorbent atoms and tube atoms. The potential energy is given by:

3 $$E=4\epsilon \left[{\left(\frac{{\rm{\sigma }}}{r}\right)}^{12}-{\left(\frac{{\rm{\sigma }}}{r}\right)}^{6}\right]r < {r}_{c}$$

Where $\epsilon$, the depth of the potential well and ${\rm{\sigma }}$, the zero-potential distance, are uniquely assigned to each atom based on SPCE (for water), and OPLS-aa forcefield (benzoic acid and CNT) and geometrically averaged for the pair in question. The sum of the pair-wise energy of all adsorbent-CNT atom pairs separated by a distance $r$ within the cutoff distance ${r}_{c}$, taken here as 12 Å, is the binding potential. Error margins are calculated based on 10 sample time instances, chosen to be 50 ps apart and situated at the end of the production run.

Theoretical pair energies were first obtained for (1) benzoic acid and CNT and (2) water and CNT at 10 sample instances. This allows for a statistically reasonable first approximation into the energetics of benzoic acid adsorption and the attendant competition from water. For example, for the first potential above (benzoic acid and CNT), the per molecule energy of benzoic acid with CNT is given by:

4 $$({E}_{\begin{array}{c}benzoicacid\\ tube\end{array}}-({E}_{\begin{array}{c}benzoicacid\\ benzoicacid\end{array}}+{E}_{\begin{array}{c}tube\\ tube\end{array}}))/coun{t}_{benzoicacid}.$$

**Figure 6:**
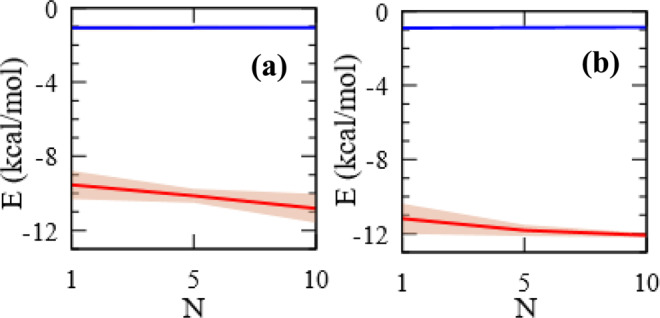
Adsorption potential with respect to N number of benzoic acid molecules closest to CNT (10,10), (**a**), and closest to CNT (40,40), (**b**); benzoic acid in red, and water in blue; error margins shown by the shaded regions; curves shown from tube surface; calculation cutoff for water molecules explained in main text.

The radial boundaries set are selected to exclude all benzoic acid molecules but for 10, 5, or 1 benzoic acid molecules closest to tube. The potentials thus calculated are compared with those of water molecules within the same boundaries. Regardless of curvature, the binding of benzoic acid is an order of magnitude more favored than that of water (Fig. 6). We further investigated the effect of tube bundling on the adsorption potential of benzoic acid. For this purpose, we computationally compared the potential at the adsorbed state of a benzoic molecule in the vicinity of an individual multi-walled tube with that of a benzoic acid molecule in the vicinity of a bundle of single walled tubes.

**Figure 7:**
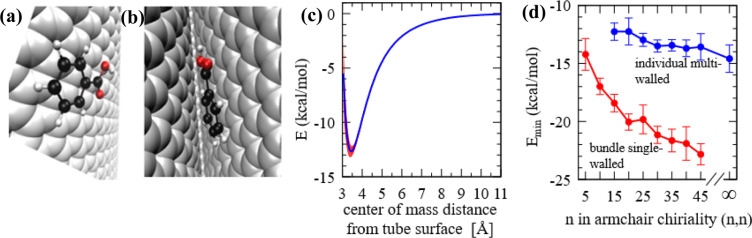
(**a**) Snapshot of an adsorbed benzoic acid molecule on carbon nanotube exterior on tube surface and, (**b**) inside groove of a bundle. These images were generated by using the open software code VMD^41^. (**c**) sample center of mass distance-potential relationship: systematic operation whereby an adsorbed benzoic acid molecule is removed from the surface of an individual multiwalled tube of chirality (15,15) in very small increments controlling for orientation; error margin represented by the shaded region in red, (**d**) minimum potential as a function of chirality for individual multiwalled nanotubes of outer chirality (n,n), and 3-tube bundles of the identical chirality (n,n).

For this simulation, a single benzoic acid molecule was allowed to adsorb separately on a three-walled tube with outer chirality of (15,15) [$r=10.16\,{\rm{\AA }}$], and on a three-tube bundle of individual tubes of chirality (15,15). We assumed the recommended inter-tube and inter-wall distance of 3.4 Å^39^. Walls beyond 3 were not considered as they are outside the LJ influence radius ${r}_{c}\,=12\,{\rm{\AA }}$ from the adsorbing benzoic acid molecule. On the surface of the multi-walled nanotube, the molecule’s trajectory shows that, on average, it orients optimally with the ring normal vector forming an 89.7° angle with the tube longitudinal axis and a 2.0° deviation from the radial vector to the center of mass. This is indicative of a face-flat adsorption preference (Fig. 7a). Bundles of the single walled tubes, on the other hand, afforded a more energetically favorable adsorption site in the grooves (Fig. 7b) where the adsorbing molecule consistently locates itself regardless of starting position during the simulation setup, and where the potential is calculated to be −18.42 ± 0.76 kcal/mol. This is a considerably greater adsorption potential compared to −12.26 ± 0.74 kcal/mol that was calculated on the multi-walled carbon nanotube. The ratio of these two adsorption potential values is ~ 1.5.

To put this in a larger curvature-dependent context, the binding potential calculations were repeated for a range of chiralities in both the individual multi-walled and bundle single-walled arrangements. Following the same inter-wall distance requirement of 3.4 Å, the smallest 3-walled nanotube that can be modeled is one with an outer radius $r=10.16\,{\rm{\AA }}$ [chirality (15,15)]. A sample distance dependence of surface potential is shown in Fig. 7c. The limiting case of a 3-walled graphene, where radius/chirality index goes to $\infty$, is shown in Fig. 7d. It is noteworthy that the binding potential on single walled tube surfaces shows a steadier and steeper decrease from −9 kcal/mol to −13.5 kcal/mol within the same range of chiralities (data not shown). The corresponding case on the surface of multiwalled tubes is, however, governed by an overall balancing effect of inner walls with regard to pair distances contributing to the ${\left(\frac{{\rm{\sigma }}}{r}\right)}^{6}$ attractive part and the ${\left(\frac{{\rm{\sigma }}}{r}\right)}^{12}$ repulsive part of the LJ potential, and thus is not as steep. This is shown in Fig. 7d. More importantly, bundles made of single-walled nanotubes show a consistently greater attractive preference across the different radii investigated compared to individual multiwalled counterparts.

## Conclusions

In conclusion, this report presents a synergetic experimental and MD simulations-based investigation of adsorption of benzoic acid, a model molecule of emerging water contaminants, on carbon nanotubes. Binding potentials for benzoic acid, calculated from simulations on grooves of SWNT bundles were found to be significantly higher than those on surfaces of MWNTs. The simulations further revealed that the adsorbing benzoic acid molecule consistently locates itself in the grooves of the bundles regardless of its starting position. These are perhaps the key reasons for higher adsorption capacity of benzoic acid on the SWNTs as observed during the experiments compared to other high surface area adsorbents reported earlier. The simulations also found that the binding of benzoic acid to CNTs is an order of magnitude more favorable than that of water. It is useful to reiterate that although the present study uses benzoic acid as a model contaminant, we have previously^40^ reported on the CNT adsorption behavior of a class of aromatic organic contaminants with varied aromatic ring multiplicity and structure of functional group in a non-dissociative environment similar to that in the present work. While the existence of more aromatic rings corresponds to more adsorptive aromatic-aromatic interactions, determination of the effect of the nature of functional group requires a more specific accounting of the composition of attractive and repulsive adsorbent-adsorbate atom pairs within the van der Waals influence separation distance^41^. All these findings therefore underscore the importance of structure as well as assembly of these carbon nanomaterials as it relates to contaminant adsorption and provides new insights into developing these materials for large-scale water purification applications. Future studies may examine closely the pore size distribution of these two CNTs and how Fe impurity in the Fe-SWNTs affect the adsorption process to further understand the adsorption mechanisms of benzoic acid on different types of CNTs.

## Supplemenatry information

Supplemenatry information.
