# Supplementary material for: High Adsorption of Benzoic Acid on Single Walled Carbon Nanotube Bundles
Source: Sci Rep. 2020 Jun 19;10:10013. doi: 10.1038/s41598-020-66871-4 (PMC7305125; doi:10.1038/s41598-020-66871-4)
Supplement: Supplementary file 1 — Supplemenatry information. [file 41598_2020_66871_MOESM1_ESM.pdf]

Supplementary Information

High Adsorption of Benzoic Acid on Single Walled Carbon Nanotube Bundles

Shifan Li[1], Thushani DeSilva[2], Iskinder Arsano[3], Dinuka Gallaba[2], Robinson Karunanithy[2], Milinda Wasala[2], Xianfeng Zhang[2], Poopalasingam Sivakumar[2], Aldo Migone[2], Mesfin Tsige[3*], Xingmao Ma[1*], Saikat Talapatra[2*]
[1]Zachry Department of Civil and Environmental Engineering, Texas A&M University, College Station, TX-77843
[2]Department of Physics, Southern Illinois University Carbondale, IL-62901
[3]Department of Polymer Science, University of Akron, Akron, Ohio-44325

Pore size distribution

The pore size distribution for typical Fe-SWNT samples were determined from N_2 desorption (77K) data from ASPA2020 sorption analyzer. The method used in the calculation is mainly incorporated from Barrett, Jouyner and Halenda (BJH) method [S1] with some corrections to the Kelvin equation (Faas Correction). This method uses cylindrical pore approximation. It is assumed that in a certain relative pressure range (p/p0 < 0.98) the capillary condensation takes place in pores. Then at the desorption branch of the isotherm, the pores are emptied and it is represented as the hysteresis in the isotherm. This is used to find the amount of adsorbate lost in the process, which resembles the average size of pores emptied. The remaining layer of the adsorbate on the walls of the pore will evaporate in further decrease of the pressure. The pore size distribution is presented in **Figure S1**. It was found that the samples had a broad pore size distribution with most of the pores in the meso (2–50 nm) to macro (>50nm) pore range. The absence of microspores with pore size < (2nm) in the distribution manifests that the tubes are closed ended as well as there is no adsorption in the interstitial channels.

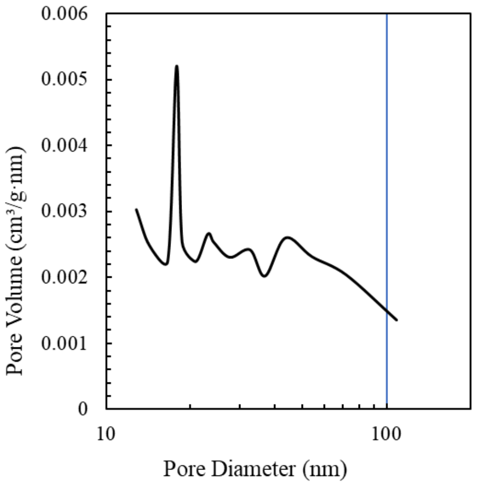

Figure S1. Pore size distribution of Fe-SWNTs.

Langmuir model fitting (Linear scale)

We estimated the maximum adsorption capacity of benzoic acid on G-MWNT and Fe-SWNT by fitting the data with Langmuir Isotherm equation [S2]. The results of this fit in shown in linear scale here (**Figure S2**). This data corresponds to figure 4a. in the main manuscript.

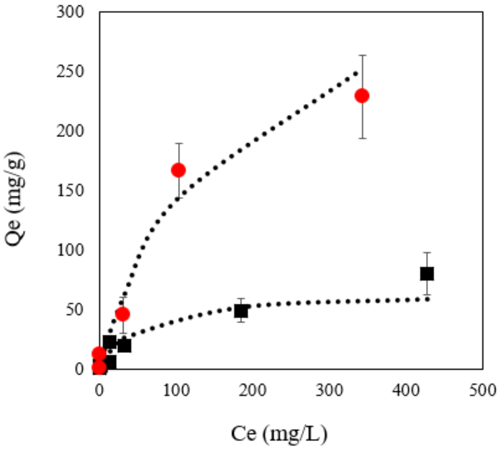

Figure S2. Adsorption of benzoic acid on commercially available G-MWNT (square) and as produced Fe-SWNT (circle). Langmuir fits (dotted line).

Point of zero charge determination

To gain more insights into the electrostatic properties of carbon materials used in this study, we have used titration [S3] to find the point of zero charge for both carbon materials and their relative charges at different pH values. The charge density curve as shown below (**Figure S3**) was calculated by different NaOH consumption by carbon

nanotubes and the blank. The results suggest that the point of zero charge of G-MWNTs was around 2.1 and the point of zero charge of SWNTs was around 9.25.

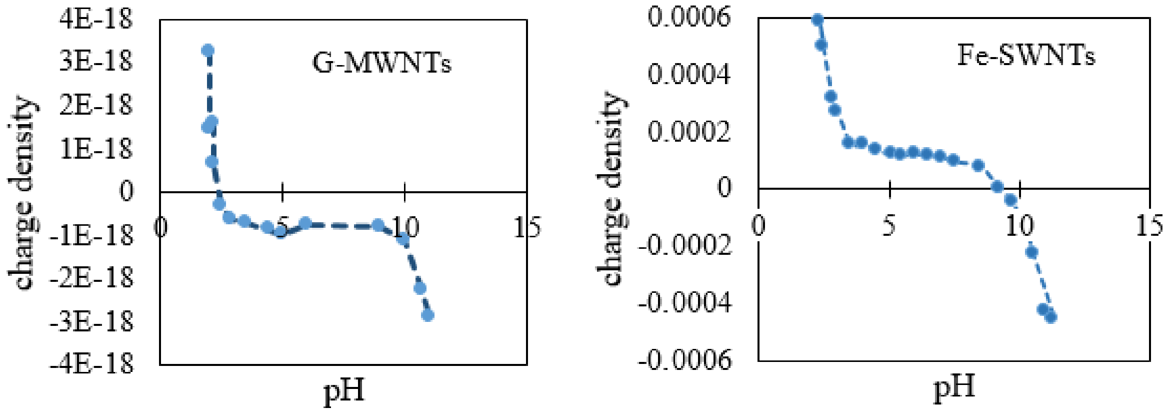

Figure S3. Titration curves of G-MWNT and Fe-SWNT.

References

S1. Barrett, E. P., Joyner, L. G., & Halenda, P. P. (1951). The determination of pore volume and area distributions in porous substances. I. Computations from nitrogen isotherms. Journal of the American Chemical society, 73(1), 373-380.

S2. Li, X., Pignatello, J. J., Wang, Y., Xing, B. (2013). New insight into adsorption mechanism of ionizable compounds on carbon nanotubes. Environ Sci. Technol., 47(15), 8334-8341.

S3. Lutzenkirchen, J., Preocanin, T., Kovacevic, D., Tomisic, V., Lovgren, L., Kallay, N. (2012). Potentiometric titraions as a tool for surface charge determination. Croatica Chemica Acta., 85(4), 391-471.
